# Effects of Temperature and X-rays on Plastic Scintillating Fiber and Infrared Optical Fiber

**DOI:** 10.3390/s150511012

**Published:** 2015-05-11

**Authors:** Bongsoo Lee, Sang Hun Shin, Kyoung Won Jang, Wook Jae Yoo

**Affiliations:** School of Biomedical Engineering, College of Biomedical & Health Science, BK21 Plus Research Institute of Biomedical Engineering, Konkuk University, 268 Chungwon-daero, Chungju-si, Chungcheongbuk-do 380-701, Korea; E-Mails: bslee@kku.ac.kr (B.L.); shshin9431@gmail.com (S.H.S.); kko988@kku.ac.kr (K.W.J.)

**Keywords:** fiber-optic sensor, plastic scintillating fiber, infrared optical fiber, X-ray, temperature

## Abstract

In this study, we have studied the effects of temperature and X-ray energy variations on the light output signals from two different fiber-optic sensors, a fiber-optic dosimeter (FOD) based on a BCF-12 as a plastic scintillating fiber (PSF) and a fiber-optic thermometer (FOT) using a silver halide optical fiber as an infrared optical fiber (IR fiber). During X-ray beam irradiation, the scintillating light and IR signals were measured simultaneously using a dosimeter probe of the FOD and a thermometer probe of the FOT. The probes were placed in a beaker with water on the center of a hotplate, under variation of the tube potential of a digital radiography system or the temperature of the water in the beaker. From the experimental results, in the case of the PSF, the scintillator light output at the given tube potential decreased as the temperature increased in the temperature range from 25 to 60 °C. We demonstrated that commonly used BCF-12 has a significant temperature dependence of −0.263 ± 0.028%/°C in the clinical temperature range. Next, in the case of the IR fiber, the intensity of the IR signal was almost uniform at each temperature regardless of the tube potential range from 50 to 150 kVp. Therefore, we also demonstrated that the X-ray beam with an energy range used in diagnostic radiology does not affect the IR signals transmitted via a silver halide optical fiber.

## 1. Introduction

Optical fiber-based sensors offer many advantages over conventional sensors, including small sensing volume, light weight, good flexibility, real-time and remote sensing, absence of electrical wires in the sensing probe, and immunity to electromagnetic interference (EMI) and radiofrequency interference (RFI). Accordingly, various fiber-optic sensors have been investigated and developed as promising candidates to overcome the shortcomings of existing physical, chemical, and biological sensors. In particular, for medical usage, fiber-optic dosimeters (FODs) and fiber-optic thermometers (FOTs) have been investigated to obtain radiation and temperature information, respectively.

FODs have been developed in conjunction with various scintillators to distinguish many kinds of radiation and to measure the related energies and dose distribution. Over the past 20 years, fiber-coupled organic scintillators including plastic scintillating fibers (PSFs) have been traditionally used for *in situ* dose measurement because of their desirable dosimetric qualities, such as a highly water equivalent characteristic, fast decay time, high spatial resolution, minimal perturbation of the radiation field, and the capability of providing real-time dose monitoring for *in vivo* dosimetry [1,2,3,4,5,6,7,8,9,10,11,12,13]. Recently, however, there has been considerable research that proves temperature affects the scintillator light output [14,15,16]. Buranurak *et al.* carried out an experimental study on temperature variation as a source of uncertainty in medical fiber-coupled organic plastic scintillator dosimetry [14]. For the BCF-12 and BCF-60, PSFs manufactured by Saint-Gobain Ceramic & Plastics, temperature coefficients of −0.15 ± 0.01%/K and −0.55 ± 0.04%/K, respectively, were identified. The temperature dependence of BCF plastic scintillation detectors was also described by Wootton and Beddar [15]. In their experiment, BCF-12 exhibited a 0.09% decrease and BCF-60 a 0.50% decrease in measured dose per °C increase, relative to the dose measured at 22 °C.

Next, in the cases of FOTs, they normally use a silver halide optical fiber as an infrared optical fiber (IR fiber) to measure temperature by transmitting IR signals emitted from a heat source [17,18,19,20,21,22]. As a non-contact temperature sensor, those based on IR fibers have been fabricated to use in examination or treatment rooms where magnetic resonance imaging (MRI) or radiofrequency ablation (RFA) devices are installed [20,21]. Previous reports demonstrated that IR fiber-based FOTs have immunity to high EMI and RFI. However, it is also necessary to consider the effects of the radiation on the light output signals from FOTs based on IR fibers for application in hybrid medical devices, such as combined positron emission tomography (PET)-computed tomography (CT) systems and PET-MR imaging systems.

We have therefore studied the effects of temperature and X-ray energy variation on each light output signal generated from a FOD based on a PSF and a FOT using an IR fiber, respectively. In this study, two different types of sensing probes, which can produce scintillating light or transmit an IR signal, were fabricated to evaluate various non-ideal characteristics of the PSF and IR fiber. Using the fabricated two sensing probes, we measured the scintillating light generated from a PSF and the IR signal transmitted via an IR fiber during beam irradiation under different X-ray energy or temperature and compared the experimental results with the radiation dose and temperature obtained using a conventional dosimeter and a thermometer, respectively.

## 2. Materials and Experimental Setup

### 2.1. Fabrication of a Fiber-Optic Dosimeter and a Fiber-Optic Thermometer

**Figure 1:**
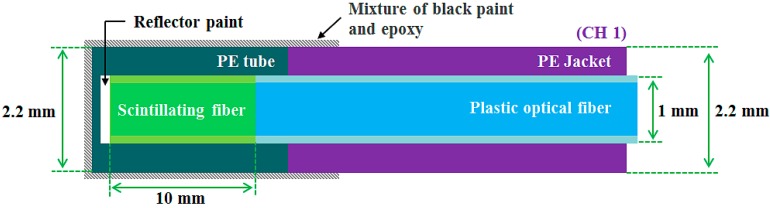
Internal structure of the dosimeter probe.

**Figure 2:**
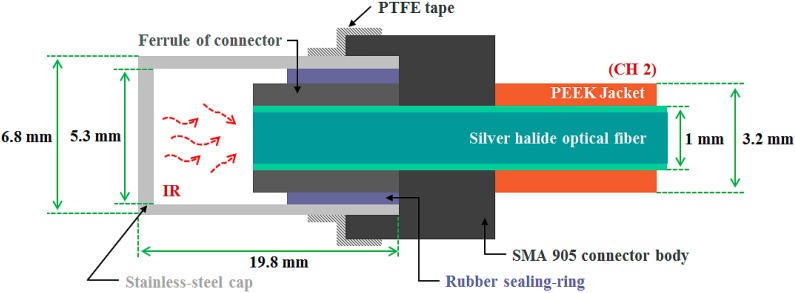
Internal structure of the thermometer probe.

The main study concerns the impact of temperature or X-ray energy variation on the light output signals from a fiber-optic sensor based on a PSF or an IR fiber. Figure 1 and Figure 2 show the internal structures of the two different types of sensing probes, which can produce scintillating light and transmit an IR signal, respectively.

First, we fabricated a typical dosimeter probe (CH 1) of a FOD to measure scintillating light signals induced by an X-ray beam. As a sensing element of a dosimeter probe, a PSF (BCF-12, Saint-Gobain Ceramic & Plastics, Hiram, OH, USA) was used to produce scintillating light with an emission peak in the blue range owing to X-rays. As an organic scintillator, BCF-12 is the most commonly used PSF in FOD [5,6,7,8,9,10,11,12,13,14,15]. This PSF has a core/single-clad structure and a cylindrical shape with an outer diameter of 1 mm. The thickness of the cladding is approximately 3% of the PSF diameter. The core of the PSF is synthesized with polystyrene (PS) and fluorescent dopants and the cladding is made of polymethylmethacrylate (PMMA). Accordingly, the refractive indices of the core and the cladding are 1.60 and 1.49, respectively, and the numerical aperture (NA) is about 0.58. The emission peak and the decay time of the PSF are 435 nm and 2.7 ns, respectively, and the operating temperature range is from −20 to 50 °C [23]. In order to transmit scintillating light, the PSF was coupled to a plastic optical fiber (POF: GH-4001, Mitsubishi Rayon, Tokyo, Japan). This optical fiber is multi-modal and has a step refractive index profile. The diameter of the core is 0.98 mm and with the cladding the diameter is 1 mm. The POF consists of a PMMA core with a refractive index of 1.49 and fluorinated polymer-based cladding with a refractive index of 1.402; thereby the NA of this POF is about 0.5. The maximum transmission loss of the POF is 170 dB/km when used with 650 nm collimated light and the operating temperature is from −55 to 85 °C [24]. The POF is covered by a black polyethylene (PE) jacket with an outer diameter of 2.2 mm to block external light noise and to protect the POF from ambient contamination. Figure 1 illustrates the internal structure of the dosimeter probe. In fabricating the typical dosimeter probe of the FOD, first, both ends of the PSF and POF, respectively, were polished with various types of lapping films (LFG series, Thorlabs, Newton, NJ, USA). Second, the proximal end of the 10 mm length PSF was mechanically connected to the distal end of the POF with a length of 6 m for transmitting scintillating light. Third, the distal end of the PSF was coated with a titanium dioxide (TiO_2_)-based reflector paint (BC-620, Saint-Gobain Ceramic & Plastics) to increase the collection efficiency of the scintillating light. Fourth, the entire PSF was covered with a black PE tube and the outer surface of the dosimeter probe was additionally coated by a mixture of a black acrylic paint (Alpha Artist Acrylic Colors, Alpha Art Materials, Seoul, Korea) and an optical epoxy (DP-100 Plus, 3M, St. Paul, MN, USA) with a mass ratio of 3:7 to prevent the ingress of external light. Fifth, a subminiature type A (SMA) 905 connector was installed at the proximal end of the POF to enable the dosimeter probe to be connected with a photon-counting device. Lastly, the FOD system was completed by connecting the dosimeter probe with a photon-counting device, a multi-pixel photon counter (MPPC) module (C10751-03, Hamamatsu Photonics, Hamamatsu, Japan) with an internal MPPC device (S10362-11-100U, Hamamatsu Photonics) that has high sensitivity in a spectral response range from 320 to 900 nm and a peak sensitivity wavelength of 440 nm [7]. The output signals from the MPPC module with a threshold level of 0.5 photon equivalent (p.e.) and a gate time of 1 ms are transmitted via a universal serial bus (USB) line to a laptop running emulation software. During the beam irradiation, we obtained the total counts of the scintillating light generated from the PSF in the dosimeter probe.

Next, to measure IR signals induced by temperature, an embedded-type thermometer probe (CH 2) of a FOT was also constructed. As an IR fiber, we selected a silver halide optical fiber (PIR 900/1000, Art Photonics, Berlin, Germany) that can serve as a sensing element and an IR waveguide at the same time [17,18,19,20,21,22]. Diameters are 0.9 mm for the core only and 1 mm including the cladding. The refractive indices of the core and the cladding are 2.15 and 2.13, respectively, and the NA is about 0.25. This IR fiber is produced with silver bromide chloride (AgBrCl) polycrystalline, and the jacket is made of polyether ether ketone (PEEK) polymer. The silver halide optical fiber enables the transmission of mid-IR range from 4 to 18 μm. This IR fiber is also very flexible and durable over a temperature range from −200 to 250 °C and its melting point is 415 °C [25]. Figure 2 shows the internal structure of the thermometer probe, which is composed of a stainless-steel cap, a SMA 905 connector, a rubber sealing-ring, and a silver halide optical fiber with a length of 1 m. The stainless-steel cap was employed to protect the end surface of the silver halide optical fiber from harsh environments and to circumvent any emissivity effects of the measured heat source. The inner gap at the coupling interface between the stainless-steel cap and the ferrule of the SMA 905 connector was sealed by a rubber sealing-ring. In addition, the outer surface of the coupling interface between the stainless-steel cap and the SMA 905 connector body was also sealed with polytetrafluoroethylene (PTFE) tape (*i.e.*, Teflon). Unlike general non-contact types of FOTs [17,18,19,20,21], this type of thermometer probe can be directly embedded in a water environment and also can measure inner temperature in real time [22]. The completed thermometer probe was connected to a thermopile (A2TPMI334OAA060, Perkin Elmer, Wiesbaden, Germany) with a sensing range from 2 to 22 μm and an amplifier system [21,22]. The output voltage signals from the thermopile-amplifier system are transmitted to a data acquisition (DAQ) board (USB-6008, National Instruments, Seoul, Korea) and a laptop running the program LabVIEW (NI LabVIEW™ 2013, National Instruments).

### 2.2. Experimental Setup Using an X-ray System and a Hotplate

**Figure 3:**
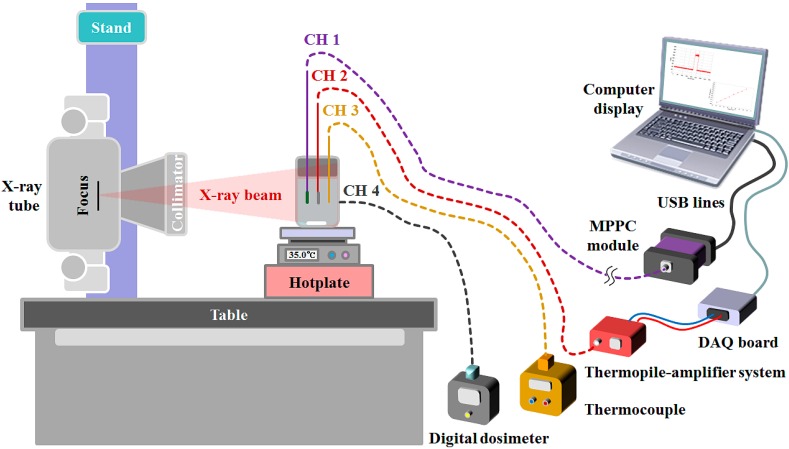
Whole experimental setup using a DR system and a hotplate.

**Figure 4:**
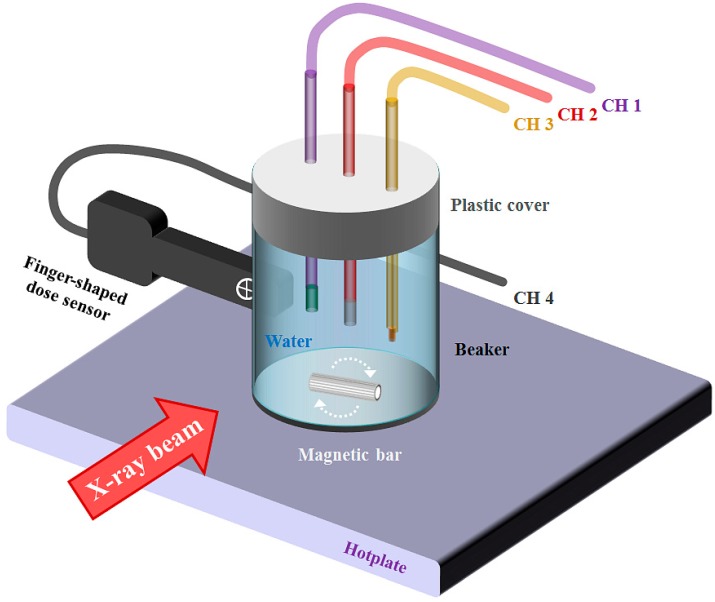
Detailed experimental setup using a hotplate with a built-in magnetic stirrer, a beaker of water and four different kinds of sensing probes.

In order to measure the effects of the temperature or X-ray energy variation on the light output signals from the PSF or IR fiber, the laboratory tests were carried out using an experimental setup consisting of an X-ray tube (Rotanode™ E7252X, Toshiba Electron Tubes & Devices, Saitama, Japan) of a digital radiography (DR) system (Clear Vision DR 7000F, JPI Healthcare, Osong, Korea) [7] and a hotplate with a built-in magnetic stirrer (REXIM RS-6DR, AS-ONE, Osaka, Japan), as illustrated in Figure 3 and Figure 4.

In general, X-rays emitted from a DR system for diagnostic radiology are Bremsstrahlung radiation. Accordingly, to correct slightly different dose values of each X-ray beam with an identical tube potential, we used a semiconductor dosimeter (SCD), a finger-shaped dose sensor probe (CH 4), connected to a digital dosimeter (Pehamed, ALPHA plus, Sulzbach, Germany). The dose values measured by using the SCD are related to the light output signals of the FOT as well as the FOD. In the case of the IR output signals from the FOT, they were compared with the *in situ* temperature values directly obtained by using a K-type thermocouple probe (CH 3) of a thermometer (54II thermometer, Fluke, Everett, WA, USA) immersed in the water, as shown in Figure 3. Through random and repeated experiments, we measured the scintillating light and IR signals simultaneously according to the tube potential (kVp) of the X-ray tube and the temperature of the water.

A beaker of water was placed on the center of the hotplate and three different types of sensing probes (CHs 1~3) were held and inserted into the water through a plastic cover on the beaker. In the case of the SCD (CH 4), it was installed beside the beaker, as shown in Figure 4 [15]. For this experiment, the X-ray tube was rotated to irradiate the sensing probes in the water perpendicularly. As exposure parameters, the values of the current-time product (tube current × irradiation time), focus-to-surface distance (FSD) and irradiation field size were fixed at 5 mA∙s (50 mA × 100 ms), 70 cm, and 20 × 20 cm^2^, respectively. When the temperature of the water was maintained at the specific desired temperature controlled by the hotplate with a built-in magnetic stirrer, four sensing probes were irradiated by X-ray beams with different tube potentials. During beam irradiation, we measured the scintillating light generated from the dosimeter probe and the IR signal generated from the thermometer probe, simultaneously.

## 3. Results

### 3.1. Effects of the Temperature Variations on the Light Output Signals from the PSF

In this study, we first measured the total counts of the scintillating light signals at a constant temperature while varying the tube potential in a range from 50 to 150 kVp. Here, the temperature of the water was changed in the clinical temperature range from 25 to 60 °C and maintained at the specific desired temperature with an uncertainty of ± 0.5 °C until achieving thermal equilibrium. Every experiment was carried out at a controlled room temperature of 25 ± 1 °C and all data (*i.e.*, counts) of the FOD were corrected by using absolute calibration factors of each X-ray beam at the given tube potential.

**Figure 5:**
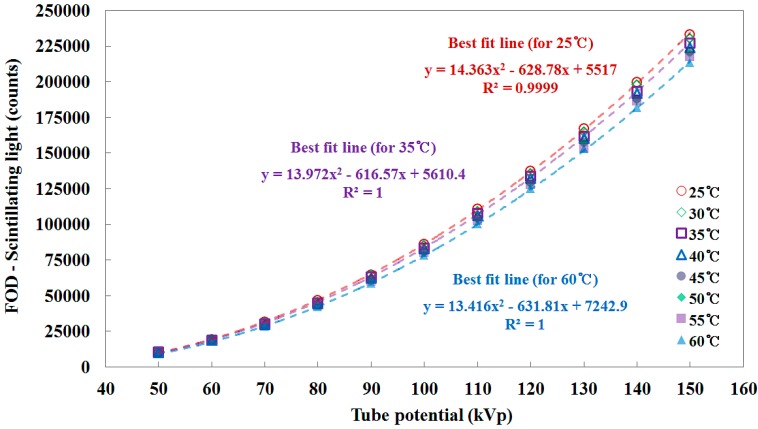
Total counts of the scintillating light signals measured by the FOD according to the tube potential under changing temperature of water.

Various experimental studies on the plastic scintillator response to low-energy photon beam have been carried out, and it was reported that the plastic scintillators have non-linearity in their scintillation response to low-energy X-rays in the range used for diagnostic radiology [4,6,7,26,27,28]. From the present experimental results, the total counts of the FOD increased with a quadratic equation according to the increase of the tube potential related to the X-ray energy, as shown in Figure 5. This is consistent with results obtained by using FODs in previous studies. However, the scintillator light output at the given tube potential decreased as the temperature increased in the temperature range from 25 to 60 °C [14,15,16].

**Figure 6:**
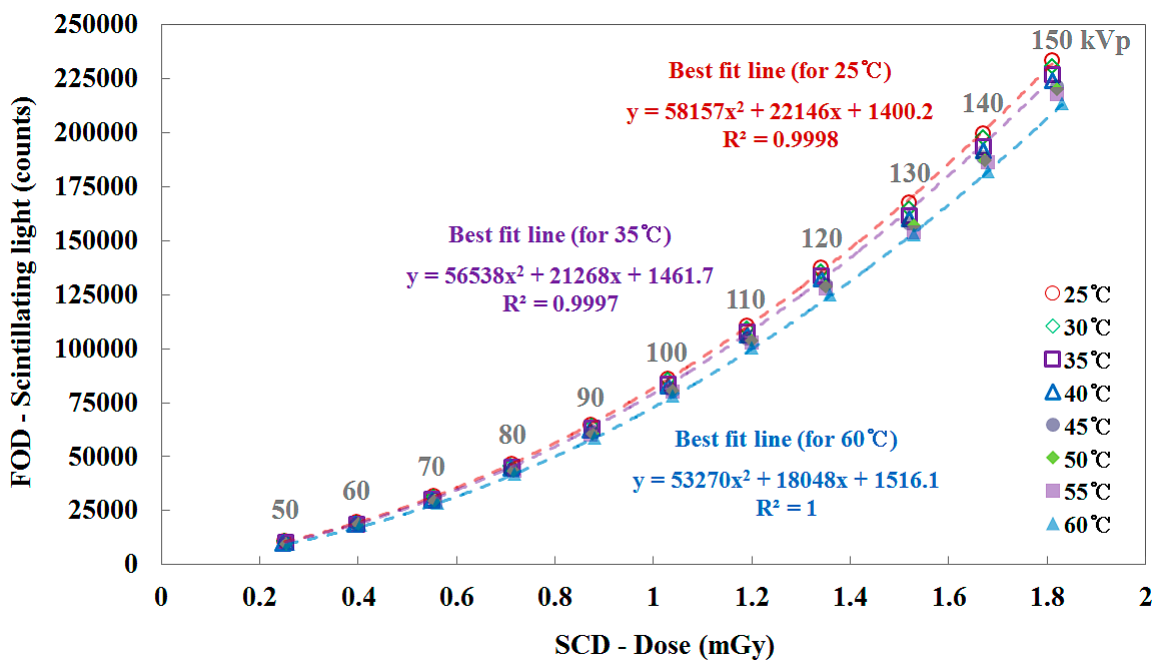
Relationship between the total counts of the FOD and the X-ray dose of the SCD as a function of the tube potential.

Figure 6 shows the relationship between the total counts of the scintillating light signals measured by the FOD and the X-ray doses measured by using the SCD as a function of the tube potential. Over the tube potential range of interest in diagnostic radiology, the total counts of the FOD increased with increasing X-ray dose due to the tube potential. In this test, it was found that the total counts curve has a gentle slope with increasing temperature and the difference between the total counts curve for 25 °C (*i.e.*, reference curve) and each curve for other temperature conditions gradually increases in accordance with the temperature rise. When the internal temperature of the beaker was higher than 45 °C, the outside temperature near the side of the beaker reached about 36 °C. In the case of the SCD, its operating temperature range is from 15 to 35 °C; consequently, the SCD had an over-response at a constant tube potential when its ambient temperature was higher than the maximum operating temperature (35 °C), as shown in Figure 6.

**Figure 7:**
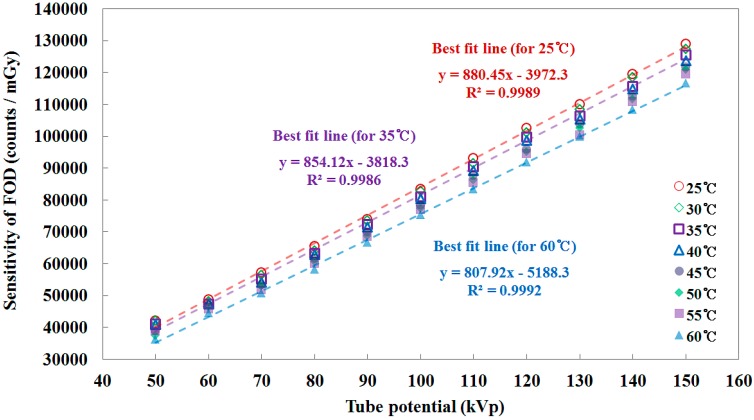
Sensitivity of the FOD as a function of the tube potential.

Figure 7 plots the sensitivity of the FOD, in units of counts/mGy, as a function of the tube potential and it was calculated using the X-ray dose values obtained by the SCD [3,9]. The sensitivity of the FOD increased linearly with 0.678 ± 0.008%/kVp across the tube potential which is related to the X-ray dose. As can be seen in Figure 7, the sensitivity of the FOD at the given tube potential also decreased according to the increase of the temperature. The mathematical forms of each best fit line for 25, 35, and 60 °C are presented in Figure 5, Figure 6 and Figure 7. The square of the correlation coefficient (R^2^), which refers to the level of agreement between the measured data and the fitting line, are also provided.

**Figure 8:**
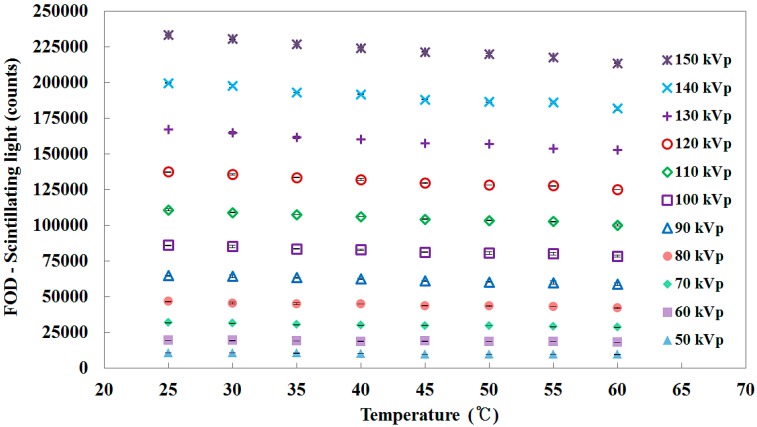
Total counts of the scintillating light signals measured by using the FOD according to the temperature variation of the water at each tube potential.

**Figure 9:**
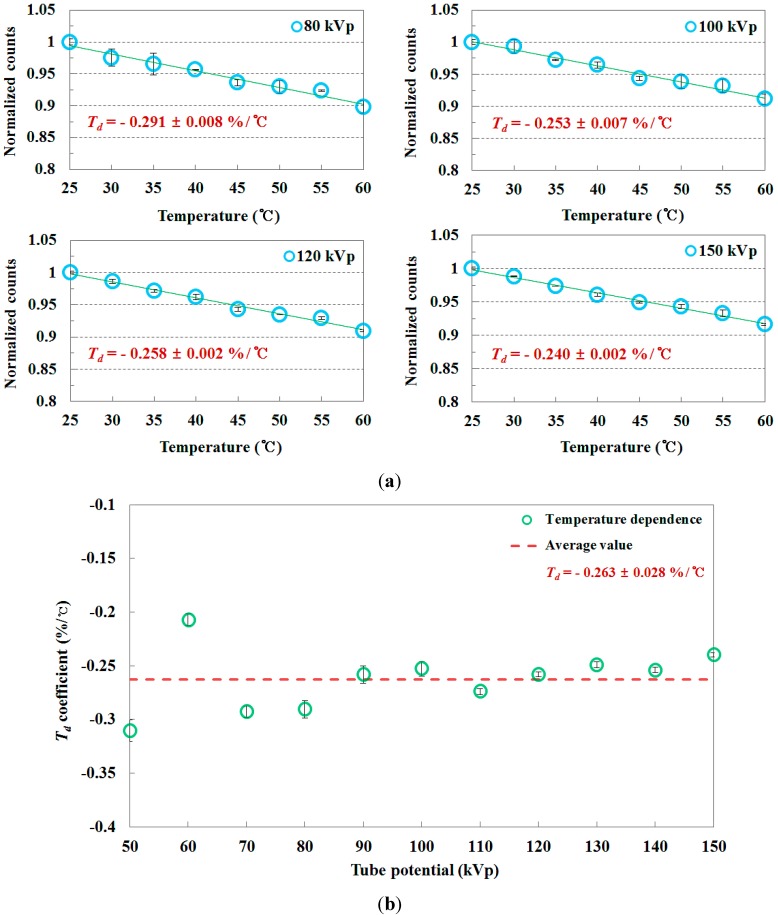
(**a**) Relationship between the temperature of the water and the normalized counts of the FOD when the tube potentials are 80, 100, 120, and 150 kVp, respectively; (**b**) *T_d_* coefficients of the PSF at each tube potential.

Figure 8 shows the total counts of the scintillating light signals measured by using the FOD according to the temperature variation of the water. Because the amount of scintillator light depends on X-ray energy, the total counts of the scintillating light signals at the given temperature increase as the tube potential increases, as shown in Figure 8. According to the temperature variation, the total counts of the scintillating light signals are also changed with an almost identical temperature dependence (*T_d_*) coefficient (*i.e.*, the rate of change of the total counts per temperature variation) of −0.263 ± 0.028%/°C in the tube potential range of interest in diagnostic radiology, as shown in Figure 9. In this evaluation, it is calculated that the minimum and maximum values of *T_d_* are −0.207 ± 0.006%/°C at 60 kVp and −0.310 ± 0.01%/°C at 50 kVp, respectively. In other words, although the PSF (*i.e.*, BCF-12) in the dosimeter probe has an almost identical *T_d_* coefficient as a function of X-ray dose, the total counts of the scintillating light signals change more at higher X-ray dose according to the temperature variation. In conclusion, *T_d_* is independent of energy variation although the scintillator light output is affected by both the *T_d_* and the energy dependence (*E_d_*) of the PSF. From these experimental results, we demonstrated that the *T_d_* of the PSF is significant in the clinical temperature range [14,15,16]. Fortunately, the ambient temperature of most X-ray systems is normally controlled and maintained by a thermo-hygrostat in examination or radiotherapy rooms.

### 3.2. Effects of X-ray Energy Variation on the Light Output Signals from the IR Fiber

**Figure 10:**
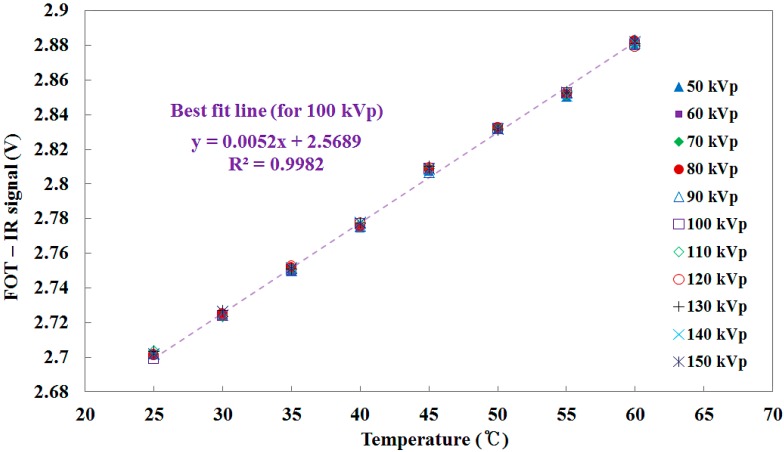
Relationship between the temperature of water and the IR signal measured by the FOT.

Next, we evaluated the effects of X-ray energy variation on the light output signals from an IR fiber. In this test, the exposure parameters were set to the same values as those of Section 3.1. Figure 10 shows the temperature of water, which was measured using a thermocouple, *versus* the output voltage of FOT as a function of the IR signal. The output voltage of the FOT increased as the temperature of water increased because the intensity of the IR signal transmitted through the IR fiber is proportional to the temperature of the heat source [17,18,19,20,21,22]. The mathematical form of the linear fit line to the curve is also presented in Figure 10 and R^2^ was found to be 0.9982. Fortunately, the IR signal at the given temperature is fairly constant even when the tube potential was changed from 50 to 150 kVp.

**Figure 11:**
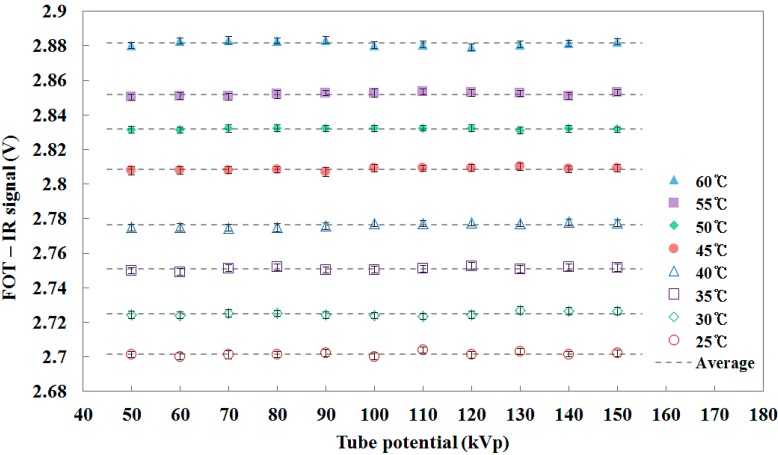
IR signal measurements of the FOT under changing tube potential conditions of the DR system.

Figure 11 shows the IR signals measured by using the FOT during X-ray beam irradiation under changing tube potential conditions. Although the tube potential was increased from 50 to 150 kVp, the intensity of the IR signal was almost uniform at each temperature and the FOT has a linear response with respect to the IR signal due to the temperature of the water. From the experimental results, we demonstrated that the intensity of the IR signal transmitted via the IR fiber (*i.e.*, silver halide optical fiber) is only affected by the temperature even when the tube potential is changed. In other words, the X-ray beam having an energy range used in diagnostic radiology does not affect the normal operation of the IR fiber. Because no effect due to the X-ray beam was observed, it was verified that reliable IR signal measurement is possible in medical and nuclear facilities with low-energy X-rays to monitor the temperature of a heat source.

## 4. Discussion

From the two main sets of experimental results, first, the scintillating light signals decreased with an almost constant *T_d_* coefficient of −0.263 ± 0.028%/°C in the tube potential range of interest in diagnostic radiology as the temperature increased in a clinical temperature range from 25 to 60 °C. Consequently, we demonstrated that a commonly used PSF, BCF-12, has a significant *T_d_* response. According to the experimental results, the *T_d_* is independent of energy variation although the scintillator light output is affected by both the *T_d_* and the *E_d_* of the PSF.

Prior to our study, Buranurak *et al.*, found that the BCF-12 and BCF-60 have *T_d_* coefficients of −0.15 ± 0.01%/K and −0.55 ± 0.04%/K, respectively, in the temperature range from 15 to 40 °C with a 50 kVp X-ray beam [14]. From the experimental results, they suggested that the *T_d_* coefficient reflects inherent property of a scintillator and it is not dependent on the radiation quality. Wootton and Beddar also studied on the *T_d_* of BCF plastic scintillation detectors [15]. In their experiments, the measured doses using the BCF-12 and the BCF-60 decreased by approximately 0.09%/°C and 0.50%/°C, respectively, when the PSFs are irradiated with 100 monitor units (MU). According to their study, BCF-60 has greater *T_d_* coefficient than BCF-12 and the generations of Cerenkov light in the PSF and optical fiber are temperature independent.

As mentioned earlier, the ambient temperature of most X-ray systems installed in examination or radiotherapy rooms is normally controlled and maintained for quality assurance (QA). Therefore, it is only necessary to consider the conversion factor of the scintillating light signals reflecting the body temperature of the patient.

Next, the intensity of the IR signal was almost uniform at each temperature regardless of the tube potential. From the experimental results, we also demonstrated that an X-ray beam having an energy range used in diagnostic radiology does not affect the IR signals transmitted through a silver halide optical fiber. It was thus verified that the FOT allows measurement of IR signals without any effect due to X-rays in medical and nuclear facilities that deal with low-energy X-rays.

## 5. Conclusion

To evaluate the influence of the temperature and X-ray energy variations on the light output signals from a PSF and an IR fiber, respectively, we fabricated two different types of sensing probes that can produce scintillating light or an IR signal. While varying the tube potential or the temperature of water, the scintillating light signals and the IR signals were measured simultaneously using the dosimeter probe of the FOD and the thermometer probe of the FOT, respectively. From the experimental results, we demonstrated that the BCF-12 has significant *T_d_* response in the clinical temperature range and the X-ray beam with an energy range used in diagnostic radiology does not affect the IR signals transmitted via a silver halide optical fiber.

Based on the results of this study, it is anticipated that the typical FOD and FOT systems will be used to reliably measure the absorbed dose and temperature information, respectively, in hybrid medical devices including PET-CT and PET-MR imaging systems. Further studies will be carried out to evaluate the influence of the variation of temperature and radiation energy on the light output signals from PSF and IR fibers using a high-energy radiation source, such as a linear accelerator (LINAC) and a Co-60 therapy unit.
